# Diagnostic value of platelet indices in COVID 19 infection: a case-control study from a single tertiary care center

**DOI:** 10.1186/s43162-022-00123-x

**Published:** 2022-04-01

**Authors:** Arundhathi Shankaralingappa, Santosh Tummidi, Thirunavukkarasu Arun Babu

**Affiliations:** 1 Department of Pathology, All India Institute of Medical Sciences (AIIMS), Mangalagiri, Andhra Pradesh India; 2 Department of Pathology, All India Institute of Medical Sciences (AIIMS), Kalyani, West Bengal India; 3 Department of Pediatrics, All India Institute of Medical Sciences (AIIMS), Mangalagiri, Andhra Pradesh India

**Keywords:** Platelets, Plateletcrit, Platelet distribution width (PDW), Mean platelet volume (MPV), COVID-19, Coronavirus

## Abstract

**Introduction:**

Platelets are not only involved in hemostasis and coagulation, but play a significant role in innate immunity and inflammatory response. Excess production of cytokines and acute phase reactants affect megakaryopoiesis resulting in the release of immature platelets from the bone marrow altering platelet indices.

**Aim:**

To study platelet indices in RT-PCR-proven COVID patients and non-COVID patients.

**Methods:**

A case-control study was conducted on 199 COVID-19 patients and 198 normal individuals. Blood samples were analyzed in an automated hematology analyzer. The platelet indices like platelet count, mean platelet volume (MPV), platelet distribution width (PDW), platelet large cell count (PLCC), and platelet large cell ratio (P-LCR) were compared among two groups.

**Results:**

Platelet count in COVID-19 patients were significantly low (p<0.01) compared to controls, and a significant number of COVID-19 patients had thrombocytopenia. Plateletcrit (PCT) was also significantly decreased in COVID-19 patients compared to non-COVID individuals. MPV, PDW, and PLCR were significantly (p<0.05) high in COVID-19 patients in comparison to controls, but was not significantly raised in a large number of cases. In contrast, there were no significant differences in platelet large cell count (PLCC) values between COVID-19 cases and non-COVID-19 controls.

**Conclusion:**

Platelet indices like platelet count, PCT, MPV, PDW, and P-LCR are significantly altered in COVID-19 infection and thereby can be used as biomarkers in COVID-19. Further research is needed to find if these simple, cost-effective parameters can be used to predict the severity and prognosis in COVID-19 infection.

## Introduction

In December 2019, the novel coronavirus disease caused by the SARS CoV-2 virus was first reported from Wuhan, Hubei province, China [1]. World Health Organization (WHO) termed the virus as 2019 novel coronavirus (2019-nCov) on 7 January 2020 and 4 days later rechristened the resultant disease as COVID-19. WHO also declared COVID-19 as a public health emergency of international concern (PHEIC) and later as a pandemic as the virus started spreading rapidly across countries [2]. As per the latest data on 1 February 2022, WHO reported 373,229,380 confirmed cases and 5,658,702 deaths worldwide with an overall mortality of 1.52% [3]. India reported 4.13 crore confirmed cases with 4.95 lakh deaths as on 1 February 2022 [4].

Platelets are not only involved in hemostasis and coagulation but play a significant role in innate immunity and inflammatory response [5]. There are many studies suggesting that platelet indices are altered in inflammatory and prothrombotic responses in numerous viral infections [6]. Platelets are activated by cytokines, and they play a pivotal role in inflammation by synthesizing proinflammatory cytokines leading to leucocyte migration and binding to endothelial cells. Large platelets are more reactive and synthesize more cytokines and thromboxane A2 since their granule contents are denser than smaller platelets. Thus, their requirement increases during the acute stage of inflammation. As a result, platelet indices are altered in inflammation. The excess production of cytokines and acute phase reactants affect megakaryopoiesis resulting in the release of small volume platelets from the bone marrow altering platelet indices [7].

Several studies proved that COVD-19 is associated with cytokine storm with release of excess amounts of cytokines like IL-1b, IFN-α, IFN-ϒ, TGF-α, IL-6, IL-12, IL-18, and IL-33 [8]. Thus, alteration in platelet indices can be expected. The platelet-related indices available from an automated hematology analyzer includes platelet count (PLT), mean platelet volume (MPV), platelet distribution width (PDW), plateletcrit (PCT), and platelet large cell ratio (P-LCR) [9]. Hence, platelet indices can serve as simple diagnostic and prognostic tool in COVID-19. This study was undertaken to determine the changes in platelet indices in COVID-19 and to compare them with non-COVID controls.

## Materials and methods

This case-control study was carried out in a tertiary care centre which was a COVID-19 care hospital located in South India. All RTPCR-positive patients admitted during the period from 1 April 2020 to 31 March 2021 were included in the study and comprised the case group. Our control group included all afebrile non-COVID patients admitted during the same period. The treatment records of all included patients were accessed from medical records department. The cases were enrolled either from outpatient or inpatient departments. Patients with known hematological diseases, those using drugs that may affect platelet functions, and pregnant women were excluded. Basic demographic details, history, examination findings, results of hemogram, and other investigations were documented. Ethical approval was obtained from the Institute ethics committee before starting the study.

Using all universal safety precautions and COVID appropriate guidelines for handling of blood samples was done at the Central Lab, Department of Pathology, where 2ml of the blood was collected in ethylene-diamine tetra-acetic acid (EDTA) vacutainer for determination of platelet indices. All the blood samples were analyzed on Yumizen H550 Horiba (HORIBA ABX SAS, YUMIZEN H550, 2017) hematology auto-analyzer. The normal ranges for the platelet count, mean platelet volume (MPV) and platelet distribution width (PDW), plateletcrit (PCT), and platelet large cell ratio (P-LCR) are 150–400 × 10^3^/uL, 7–11 fl, 0–25%, 0.22–0.24%, and 15–35%, respectively [10].

Based on previous published study, the mean platelet volume and standard deviation in COVID-19 patients were 10.57 and 10.34, respectively. With a confidence interval=95%, power of 80%, with the precision of 5%, the sample size was calculated as 156 in each group [11].

### Statistical analysis

Data was analyzed using the Statistical Package for the Social Sciences (SPSS, version 23.0; IBM, USA). Descriptive statistics were calculated for demographic data. The results are reported as the mean ± standard deviation or as the median and range. The results for categorical variables were compared using a chi-square test. The means for platelet count, MPV, PDW, PCT, and P-LCR were compared using the Student’s *t* test or ANOVA. *P* values < 0.05 was considered statistically significant.

## Results

Our study included 198 cases of non-COVID controls and 199 cases of COVID-19 cases. Among cases, 132 (66.67%) were males and 66 (33.33%) were females. Among controls, 74 (37.19%) were males and 125 (62.81%) were females. The platelet count in COVID-19 patients were significantly low (p<0.01) compared to controls. Plateletcrit (PCT) was also significantly decreased in COVID-19 patients compared to non-COVID individuals. MPV, PDW, and PLCR were significantly (p<0.05) high in COVID-19 patients in comparison to controls. In contrast, there were no significant difference in platelet large cell count (PLCC) values between COVID-19 cases and non-COVID-19 controls. The details of the comparison of platelet indices between the COVID-19 cases and non-COVID-19 controls are shown in Table 1. The comparison of platelet indices between the COVID-19 cases and non-COVID-19 controls are tabulated in Table 2. The comparison of abnormal hematological indices between the COVID-19 cases and non-COVID-19 controls are mentioned in Table 3.

**Table 1:** Demographic parameters of COVID-19 cases and non-COVID-19 controls

Parameter	Cases (***n***=198)	Controls (***n***=199)
**Age (in years)**		
**• 0–10**	**02**	**00**
**• 11–20**	**17**	**03**
**• 21–30**	**24**	**36**
**• 31–40**	**48**	**50**
**• 41–50**	**32**	**56**
**• 51–60**	**42**	**33**
**• 61–70**	**22**	**18**
**• 71–80**	**08**	**03**
**• 81–90**	**03**	**00**
**Sex**		
**• Male**	**132**	**74**
**• Female**	**66**	**125**

**Table 2:** Comparison of platelet indices between the COVID-19 cases and non-COVID-19 controls

Parameter	Cases	Controls	***P*** value
	***n***=198 (Mean ± SD)	***n***=199 (mean ± SD)	
**Age**	44.2 **±** 16.3	43.2 **±**12.8	0.480
**Platelet count**	249.8 **±** 106.2	301.0 **±** 84.6	0.000
**Plateletcrit**	0.26 **±** 0.101	0.29 **±** 0.083	0.001
**Mean platelet volume**	10.37 **±** 1.40	9.60 **±** 1.03	0.000
**Platelet distribution width**	15.83 **±** 5.92	14.44 **±** 2.83	0.003
**Platelet large cell count**	70.42 **±** 29.33	75.06 **±** 28.98	0.113
**Platelet large cell ratio**	28.97 **±** 8.19	25.65 **±** 7.41	0.000

**Table 3:** Comparison of abnormal hematological indices between the COVID-19 cases and non-COVID-19 controls

Parameter	Cases	Controls	***P*** value
	***N***=198 ***n*** (%)	***N***=199 ***n*** (%)	
**Thrombocytopenia**	19 (9.60)	7 (3.52)	**0.014**
**MPV >11fl**	16 (8.08)	48 (24.12)	**0.000**
**PDW >25%**	01 (0.50)	05 (2.51%)	**0.101**
**P-LCR >35%**	21 (10.60)	15 (7.54%)	**0.287**

## Discussion

The WHO on 11 March 2020 declared COVID-19 as a pandemic after identifying >118,000 COVID-19 cases across 114 countries [12]. COVID-19 is associated with cytokine storm with exaggerated inflammatory response characterized by release of large amounts of cytokines like IL-1b, IFN-α, IFN-γ, TGF-α, IL-6, IL-12, IL-18, and IL-33 [13]. Cytokines like IL-1, IL-6, and TNF-α play a role in thrombopoiesis. Thus, platelet indices like platelet count, PCT, MPV, PDW, P-LCC, and PLCR can be used as an inflammatory marker in any condition associated with systemic inflammation [14]. MPV and PDW were increased in sepsis and were found to be used as prognostic markers [15]. Since COVID-19 is associated with cytokine storm, these platelet indices were analyzed.

In our study, we found that platelets were low in COVID-19 cases compared to controls. MPV, PDW, and PLCR were significantly high in those with COVID-19 than non-COVID-19 controls, whereas PLCC did not show any significant difference. In another study, it was found that MPV in COVID-19 patients was significantly higher than in non-COVID patients (11.6 vs 10.5 fl) matched for platelet count which was similar to our study [16]. Whereas in another study, it was found that MPV was higher in COVID-19 patients above 65 years of age, but there was no statistically significant change in MPV in those less than 65 years of age [11]. Though many studies have proved that raised MPV serve as a marker for severity and prognosis of inflammation, Aktas et al. did not observe such phenomenon in COVID-19 [11]. An increase in MPV signifies an increase in circulating young platelets [17]. A positive correlation has been previously established between MPV and platelet activation and its function. Inflammatory cytokines regulate thrombopoiesis and MPV and reflect both prothrombotic and proinflammatory states [18].

In our study, we found that there were a significant number of COVID-19 patients with thrombocytopenia. Though MPV values were statistically significant between groups, it was not raised in a large number of COVID-19 patients. PDW and P-LCR were not significantly seen to be raised in all COVID-19 patients. A recent study found that one unit increase in MPV between first and third days of hospitalization increased mortality by 1.76 times. Thus, they emphasized that MPV can be used as an auxiliary test in predicting mortality in those suffering from COVID-19 [19]. They also found that MPV and PDW were increased in non-survivors. They stated following hypothesis to be responsible for a change in platelet count which ultimately increases MPV and PDW. Like other viruses, suppression of the bone marrow is responsible for thrombocytopenia, destruction of platelets by the immune system and aggregation of platelets in lungs leads to platelet consumption. As a compensatory mechanism, thrombocytopenia results in increased platelet production with the release of functionally active young platelets resulting in raise in MPV and PDW [19].

It was seen that another study had conflicting findings with our study with respect to MPV. MPV in COVID-19 patients was decreased compared to those with influenza. Whereas PDW showed an increase in COVID-19 patients similar to our study. PDW signifies the platelet size distribution range, and a high PDW is due to platelet destruction accompanied by the production of immature platelets. Thus, the cytokine storm encountered in COVID-19 is mainly responsible for this significant rise in PDW [20].

A Turkish study found that MPV positively correlated with COVID-19 severity [5]. Platelets play many vital roles in hemostasis, coagulation cascade, maintaining vascular integrity, neoangiogenesis, inflammatory response, innate immunity, and tumor biology. Current research has proved beyond doubt that platelets act as critical modulators of thrombosis, vascular inflammation, and in microbial infection [21]. They are also involved in inflammatory processes and cytokines like IL-3, IL-6, IL-9, IL-11, and stem cell factor induce production of megakaryocytes, which are the platelet precursors in bone marrow [22].

He et al. stated that increased PDW, MPV, and P-LCR were associated significantly with poor prognosis in COVID-19 patients [9]. Another study also concluded that MPV can serve as an important predictor and had a better predictive value than lymphocyte counts in asymptomatic COVID-19-infected children, and they also quoted that when a cutoff value of MPV was set as 8.74fl as a predictor of COVID-19, sensitivity and specificity reached 81.82% and 95%, respectively [23]. But in our study, we found significant changes in P-LCR along with MPV and PDW in COVID-19 patients as compared to normal individuals.

## Conclusion

Platelet indices like MPV, PDW, PLCC, and P-LCR are simple, cost-effective, and easily available laboratory parameters from routinely used automated hematology analyzers. These parameters were observed to be altered in COVID-19 patients in comparison with non-COVID individuals. Thus, these parameters can be used as efficient biomarkers in COVID-19. But further studies on large numbers are needed to completely determine the utility of platelet indices in COVID-19 as a prognostic marker.

## Data Availability

The datasets used and/or analyzed during the current study are available from the corresponding author on reasonable request.

## References

[1] Guo Z-D, Wang Z-Y, Zhang S-F, Li X, Li L, Li C (2020). Aerosol and surface distribution of severe acute respiratory syndrome coronavirus 2 in hospital wards, Wuhan, China, 2020. Emerging Infect Dis.

[2] Hassan S (2020). Presentation, management and pathogenesis of the SARS-CoV-2 in Children. Acta Sci Paediat.

[3] WHO Coronavirus Disease (COVID-19) Dashboard. Available at: https://covid19.who.int/. Accessed on 01 Feb 2022.

[4] Coronavirus in India: latest map and case count. Available at: https://www.covid19india.org. Accessed on 01 Feb 2022.

[5] Ozder A (2020). A novel indicator predicts 2019 novel coronavirus infection in subjects with diabetes. Diabetes Res Clin Pract.

[6] Leffondre K, Abrahamowicz M, Regeasse A, Hawker GA, Badley EM, Belzile E (2004). Statistical measures were proposed for identifying longitudinal patterns of change in quantitative health indicators. J Clin Epidemiol.

[7] Şahin M, Duru NS, Elevli M, Civilibal M (2017). Assessment of platelet parameters in children with pneumonia. J Pediatr Inf.

[8] Channappanavar R, Perlman S (2017). Pathogenic human coronavirus infections: causes and consequences of cytokine storm and immunopathology. Semin Immunopathol.

[9] He J, Wei Y, Chen J, Chen F, Gao W, Lu X (2020). Dynamic trajectory of platelet-related indicators and survival of severe COVID-19 patients. Crit Care.

[10] Budak YU, Polat M, Huysal K (2016). The use of platelet indices, plateletcrit, mean platelet volume and platelet distribution width in emergency non-traumatic abdominal surgery: a systematic review. Biochem Med (Zagreb).

[11] Aktas A, Sener K, Yilmaz N, Tunc M, Yolcu S, Aktaş A (2020). Is mean platelet volume useful for predicting the prognosis of COVID-19 diagnosed patients?. Int J Res Stud Med Health Sci.

[12] World Health Organization. WHO declares COVID-19 a pandemic (2020) Available from: https://www.who.int/dg/speeches/detail/whodirector-general-s-opening-remarks-at-the-media-briefing-on-COVID-19%2D%2D-11- march-2020 Accessed on 01 Feb 2022.

[13] Shankaralingappa A, Thirunavukkarasu AB (2021). Pathogenesis of COVID-19 and multi-system inflammatory syndrome in children. Int J Contemp Pediatr.

[14] Yolcu S, Beceren GN, Tomruk Ö, Doguç DK, Balbaloglu O (2014). Can mean platelet volume levels of trauma patients predict severity of trauma?. Platelets.

[15] Guclu E, Durmaz Y, Karabay O (2013). Effect of severe sepsis on platelet count and their indices. Afr Health Sci.

[16] Wool GD, Miller JL (2021). The impact of COVID-19 disease on platelets and coagulation. Pathobiology.

[17] Handtke S, Steil L, Palankar R, Conrad J, Cauhan S, Kraus L (2019). Role of platelet size revisited-function and protein composition of large and small platelets. Thromb Haemost.

[18] Guan WJ, Ni ZY, Hu Y, Liang WH, Ou CQ, He JX (2020). Clinical characteristics of coronavirus disease 2019 in China. N Engl J Med.

[19] Güçlü E, Kocayiğit H, Okan HD, Erkorkmaz U, Yürümez Y, Yaylacı S (2020). Effect of COVID-19 on platelet count and its indices. Rev Assoc Med Bras.

[20] Ozcelik N, Ozyurt S, Yilmaz Kara B, Gumus A, Sahin U (2021). The value of the platelet count and platelet indices in differentiation of COVID-19 and influenza pneumonia. J Med Virol.

[21] Hvas AM (2016). Platelet function in thrombosis and hemostasis. Semin Thromb Hemost.

[22] Behrens K, Alexander WS (2018). Cytokine control of megakaryopoiesis. Growth Factors.

[23] Gumus H, Demir A, Yukkaldıran A (2021). Is mean platelet volume a predictive marker for the diagnosis of COVID-19 in children?. Int J Clin Pract..

